# Sweet Talk: A Qualitative Study Exploring Attitudes towards Sugar, Sweeteners and Sweet-Tasting Foods in the United Kingdom

**DOI:** 10.3390/foods10061172

**Published:** 2021-05-24

**Authors:** Claudia S. Tang, Monica Mars, Janet James, Kees de Graaf, Katherine M. Appleton

**Affiliations:** 1 Department of Psychology, Faculty of Science and Technology, Bournemouth University, Poole House, Talbot Campus, Bournemouth BH12 5BB, UK; k.appleton@bournemouth.ac.uk; 2 Division of Human Nutrition and Health, Wageningen University & Research, Stippeneng 4, 6708 WE Wageningen, The Netherlands; monica.mars@wur.nl (M.M.); kees.degraaf@wur.nl (K.d.G.); 3 Department of Nursing Science, Faculty of Health and Social Sciences, Bournemouth University, Bournemouth House, Lansdowne Campus, Bournemouth BH1 3LH, UK; jjames@bournemouth.ac.uk

**Keywords:** sweet taste, sweetness, perceptions, focus groups, qualitative research, thematic analysis

## Abstract

Worldwide initiatives currently aim to reduce free sugar intakes, but success will depend on consumer attitudes towards sugar and the alternatives. This work aimed to explore attitudes towards sugar, sweeteners and sweet-tasting foods, towards consumption and related policies, in a sample of the general public of the UK. Focus groups and interviews were conducted with 34 adults (7 males, ages: 18–65 years). Thematic analysis identified six themes: ‘Value’ (e.g., pleasure, emotions), ‘Angle’ (e.g., disinterest), ‘Personal Relevance’ (to be concerned and/or change one’s own behavior), ‘Personal Responsibility’ (one has an active relationship with these food items), ‘Understanding’ (the acquisition, comprehension and application of information) and ‘It’s Not Up to Me’ (a passive approach, because intake is subjected to other factors). Both positive and negative attitudes towards sugar, sweeteners and sweet-tasting foods were expressed in all themes. Participants also reported varied engagement with and motivations towards all food items, with implications for intakes. Suggested challenges and potential strategies for reducing free sugar intakes highlighted the need for differing approaches. Future work should assess associations between attitudes and intakes. For greatest population benefit, evidence of the dominant attitudes in those in greatest need of reduced free sugar intakes would be of value.

## 1. Introduction

The World Health Organisation currently recommends reducing free sugar intakes to 10% daily energy intakes, with further benefits from a reduction to 5% daily energy intakes [1]. Defined as “monosaccharides and disaccharides added to foods and beverages by the manufacturer, cook or consumer, and sugars naturally present in honey, syrups, fruit juices and fruit juice concentrates” [1], free sugar intakes have been positively associated with increased prevalence of dental caries [2], cardiovascular disease [3], Type II diabetes [4] and, via the consumption of excess energy, with overweight and obesity [5].

Population levels of sugar consumption, however, lie above these recommendations. While free sugar intakes are difficult to accurately assess [6], total sugar intake in adults is estimated to range from 13.5% (Italy) to 24.6% (USA) total energy intake. Added sugar intakes are estimated to range from 7.3% (Norway) to 16.3% (USA) total energy intake, with higher estimates reported for children and adolescents (9.0% in Iceland to 18.6% in Norway) [6]. Worldwide initiatives to reduce sugar intakes focus largely on reducing the availability of sugar within the food supply through product reformulation and reduced portion sizes, and on shifting consumer purchasing towards foods lower in sugar [7,8,9,10].

Consumer food purchasing and consumption are determined by a range of factors from individual characteristics to environmental circumstances [11]. Sugar or sugar-sweetened food and beverage consumption is also determined by similar factors, including experiences of pleasure, taste and emotions [12,13,14,15,16], perceptions of health benefits [12,17,18], knowledge/lack of knowledge of health implications [12,13,15,17,19], habits [13,14,15,16,17], health concerns [13,14,16], familial, social and cultural norms [12,14,15,17] and food availability and accessibility [13,14,16,17], particularly a reliance on convenience or processed foods [13]. There is some evidence that suggests associations between sugar intakes, knowledge of the health implications of sugar consumption and attitudes toward sugar [20], although little work is available and associations have been described as ‘weak or inconsistent’ (p. 192, [20]).

Attitudes to and associations with sugar consumption are also potentially confounded by attitudes to the alternatives to sugar consumption—namely the consumption of less sweet foods, or the consumption of other sweetening agents, such as low-calorie sweeteners. Humans have an innate liking for sweet taste [21] and preferences for sweet taste remain high throughout childhood [21]. Many treat foods, even for adults, are sweet-tasting [21]. Limited work has investigated attitudes towards sweet-tasting foods independent of attitudes to sugar, and suggestions that such pleasures and treats should be forgone may be met with negativity [13,14,22,23].

Low- or no-calorie sweeteners (LNCS) may offer this desired sweet taste without the health implications of sugar. LNCS provide the pleasure of sweet taste without the energy content of sugar [24], and the safety and use of many LNCS in humans have been approved [24,25,26]. Consistent with work demonstrating benefits for weight management [27,28], LNCS use continues to be predominantly associated with concerns over bodyweight [14,16,29,30,31]. Consumers, however, also cite concerns over safety [32], poor taste [16], their artificial or chemical nature [14,16,31] and possible health implications [14,16], and similar concerns have been expressed by dietitians [23].

Despite the lack of preferred alternatives, many individuals report concerns over sugar intakes [16,31], and studies do find public support for strategies to reduce this consumption [13,15,32]. Data from the British Social Attitudes Survey on Obesity 2015 suggests support for bans on advertising and for the implementation of a tax on sugary drinks [32]. Forde and Solomon-Moore [13] found support for an information-based sugar reduction campaign among low-income consumers and Palmedo and Johnson [15] found support for a ‘sugar-sweetened beverage free zone’ in a community Health Centre. Any strategy however, will have limited effect if positive attitudes towards sugar and negative attitudes towards the alternatives prevail. Attitudes may furthermore depend on the policies implemented, and the attitudes of individuals towards those policies.

Given the limited existing research in this area and the current push to reduce free sugar intakes, further work exploring attitudes towards sugar, sweeteners and sweet foods is required. Considering the importance of differing policies toward sugar reduction for those attitudes, it is important furthermore, that this work is conducted in the context (time and location) of the intended policies. Increased understanding of current attitudes, both to the food items themselves and to relevant policies and suggestions for change would provide a strong basis for future work aiming to develop strategies for intervention and highlight areas of consumer concern. This research aimed to explore current attitudes towards sugar, sweeteners and sweet-tasting foods in a sample of the general public of the UK, including attitudes towards personal consumption and related policies.

## 2. Materials and Methods

This was an exploratory qualitative study using focus groups and interviews in a sample of consumers living in the UK.

### 2.1. Participants

Healthy adults, aged 18–65 years old and able to provide informed consent, were recruited from the south coast of England, through University contacts, community groups and flyers distributed in coffee shops and other public places. No further inclusion criteria were utilized. Minimal inclusion criteria and many recruitment strategies aimed to allow inclusion of volunteers from a range of backgrounds, providing a wide range of attitudes. Ethical approval for the study was granted by the Research Ethics Committee of Bournemouth University (ID: 29215) prior to commencement. All participants provided informed consent before participation and were compensated for their time.

### 2.2. Focus Groups and Interviews

The study used a combination of focus groups, dyadic interviews and solo interviews to generate a wide range of perspectives and understandings. Topics for discussion were considered by the researchers to be non-sensitive, hence suitable for discussion in focus groups and dyadic interviews, and focus groups were used to elicit collective and personal opinions. Interviews were conducted only where participants were unable to attend a focus group session. Data collection was undertaken from January 2018 to March 2020. During this time (April 2018), a nationwide tax on soft drinks that contain at least five grams of sugar per 100 milliliters, the ‘Soft Drinks Industry Levy’ (SDIL) was implemented across the UK [33]. Some focus groups were conducted prior to the implementation of the SDIL, while others were conducted following different time periods after this implementation, allowing collection of a wide range of attitudes. The specific time periods aimed to provide attitudes in both the short- and long-term following implementation of the SDIL.

### 2.3. Moderator Guide

A moderator guide was used to structure all focus groups and interviews. Questions focused on participants’ beliefs about sugar and sweeteners, their preferences and rationales for consumption or avoidance, their attitudes towards different sweetener terms or categories, attitudes towards sugar intake versus sweet-tasting food intake and their opinions on current and potential strategies to reduce free sugar intakes. The open-ended moderator guide was piloted prior to use in six interviews, and refined to ensure the clarity, relevance and value of each question. The refined guide was then used for all focus groups and interviews. Visual materials were presented at various time-points during each session, with the purpose of generating more discussion. These included: pictures of the sugar content of several commercial beverages in sugar cubes, in relation to current Public Health England recommendations [8,9]; examples of different categories of sweeteners [24,25,26]; examples of packaging using graphic imaging similar to that that has been used for cigarettes under The Standardized Packaging of Tobacco Products Regulations 2015 [34] and a BBC news article on the SDIL illustrated as a newspaper clipping. All visual materials and the moderator guide are provided in the Supplementary Materials.

### 2.4. Procedure

Focus groups and interviews were conducted using established methods [35,36,37]. All sessions were conducted in a semi-structured manner to encourage both personal and collective opinions. All sessions were audio-recorded for transcription and analysis. Each session lasted not more than an hour. All sessions began with an introduction and explanation on the study procedure, audio recording, confidentiality and anonymity. Three trained researchers moderated the various focus group sessions, using the refined moderator guide, but the sequence and use of each question depended on the flow of each session. Towards the end of each session, moderators asked if participants had any more thoughts on the topic that were not yet discussed. The session then continued until there was no further input from participants. By the last focus group, no new attitudes or reasons were generated and data collection for the study was concluded.

### 2.5. Data Analysis

Thematic analysis was selected for this exploratory work so as to not be theoretically-bound. Themes were identified using an inductive approach based on the explicit semantic content of the data [35]. Although a moderator guide was used during data collection, it did not serve as a coding scheme during analysis, nor was a coding scheme established; as the study was exploratory, theme formation was data-driven. Only discussions on sugar, sweeteners or sweet-tasting foods were analyzed. Discussion on attitudes or policies in countries other than the UK were also excluded, with the exception of explicit cross-country or cross-cultural comparisons.

Thematic analysis was performed based on Braun and Clarke’s six phases [38]. Moderators transcribed the audio recordings of all sessions that they conducted. All transcripts followed the orthographic style and notation system adapted from Braun and Clarke [38]. Transcripts were not sent back to participants for correction; a review on member checking did not find supporting evidence that this improved research quality in studies with a main purpose of theory development [39], and this would add to participant burden and reluctance to participate. All transcripts from all sessions were imported into qualitative data analysis software NVivo Version 12 [40] to be coded.

To address unitization, this study adopted a strategy that focused on meaning units rather than naturally given units. Initial codes were generated from each transcript by two researchers independently, and then agreed upon. This “negotiated agreement” (p. 305, [41]) after separate coding served to reconcile discrepancies in codes and unitization, to improve intercoder reliability. The principal investigator (PI) analyzed all transcripts, while four other researchers acted as the secondary coder. Codes were then grouped together by the PI to form subthemes and themes, and discussed and agreed upon by two secondary coders. All transcripts were then reviewed by the PI again to ensure no quotes were left out and to check the validity of all themes.

### 2.6. Researchers and Reflexivity

The PI and all other researchers were female with lean body weight, and the majority were involved in other projects on dietary sweetness at the time of this study. Three researchers had backgrounds in eating behavior, nutrition and the drivers of food choice and intake, and one researcher had a history of eating disorders; all of which may have had an impact on the identification and definition of themes or subthemes.

## 3. Results

### 3.1. Participants

Twenty-nine participants (24F, 5M) took part in seven focus groups, four participants took part in dyadic interviews (2F, 2M) and one participant took part in a solo interview (1F). Seventeen participants were aged 18–30 years, four participants were aged 31–40 years, four participants were aged 41–50 years, four participants were aged 51–65 years, and age for five participants was not recorded. Participants were recruited from the University student population (*N* = 11), from local workplaces, including the University (*N* = 9), among the parents of a local school (*N* = 5) and from the community, e.g., via coffee shops (*N* = 9). None of the participants reported being on a diet, having been diagnosed with diabetes or insulin resistance, or reported being intolerant or allergic to sugar, LNCS, wheat, gluten, rice, cereal or fruit. Three focus groups were undertaken from January–March 2018 before the implementation of the UK SDIL, one focus group was undertaken in July 2018 shortly after implementation of the UK SDIL, and three focus groups and all interviews were undertaken from January to March 2020.

### 3.2. Attitudes towards Sugar, Sweeteners and Sweet-Tasting Foods

**Figure 1:**
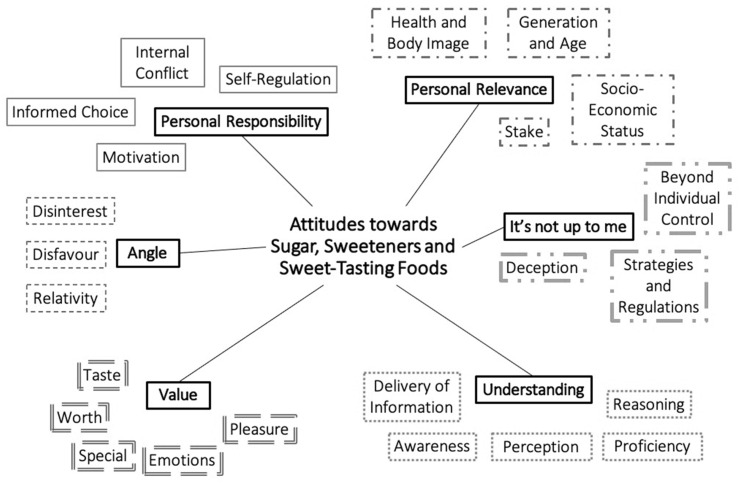
Attitudes towards sugar, sweeteners and sweet-tasting foods: themes and subthemes.

Six themes reflecting attitudes towards sugar, sweeteners and/or sweet-tasting foods were identified. These were composed of twenty-four subthemes, as shown in Figure 1, and described below. All six themes are described neutrally, because the same idea could often be expressed as present or absent, or positively and negatively by different participants. Themes are presented in no particular order, and while presented separately, interaction between themes was also possible. ‘FG’ refers to focus group, ‘DI’ refers to dyadic interview, ‘I’ refers to solo interview and ‘P’ refers to participant, e.g., [FG1, P1] labels a quote by the first participant in focus group one. Extended quotes and definitions of all themes can be found in a table format in the Supplementary Materials. All themes included attitudes towards all food items, that is sugar, sweeteners and sweet-tasting foods, however some subthemes appeared more relevant to one or two of these food items.

#### 3.2.1. Value

:: What sugar, sweeteners and sweet-tasting foods can provide.

Defined as ‘what sugar, sweeteners or sweet-tasting foods can provide’, this theme focused on the positive or negative aspects of consumption. These related to ‘taste’, ‘pleasure’, ‘special’, ‘emotions’ and ‘worth’. ‘Taste’ was considered an important factor in food choice that could not be compromised. Some participants preferred the taste of sugar while others preferred the taste of LNCS, but there was general consensus that excessive sweetness tastes unpleasant. Comments suggested preferences for high-sugar products that tasted good compared to reduced-sugar or LNCS-based versions that tasted less pleasant demonstrating the value of a pleasant taste.

‘Pleasure’ was also derived from the consumption of sugar, sweeteners and sweet-tasting foods, without reference to taste. Pleasurable experiences were suggested to prompt some participants to disregard health implications or costs; comments indicated that cravings for sugar or sweet-tasting foods should be satisfied whenever the desire came, or that the sugar content was not considered for certain foods items, such as alcoholic drinks. The appearance of LNCS packaging was also reported as unappealing, affecting acceptance and intake.

Elevated pleasure was associated with foods that were ‘Special’; foods/beverages that were considered as rewards or treats, and where intake was reserved for specific occasions, such as festive periods. These compared with food items that were considered as everyday food items; for example, dessert was considered special and valued, while a biscuit was considered regular.

Related to pleasure, in the subtheme ‘Emotions’, several statements suggested that participants valued the happiness derived from their consumption of sugar or sweet-tasting foods to the extent that if restricting intake would take away joy, they would rather experience the positive emotions. Similar positive emotions were also expressed in relation to childhood memories.

Finally, the subtheme ‘Worth’ suggested recognition of multiple but potentially conflicting benefits of sugar, sweeteners or sweet-tasting foods. Sugar was recognized to provide ‘quick energy’ and have preservative properties, while LNCS allowed limited sugar consumption for weight or diabetes management. Value for money was also an important consideration, and money was potentially more important than other considerations, hence price could drive product choice. The quality of a food/beverage product and environmental cost also mattered to some participants.

Examples quotes from this theme are:

*“Let’s say we’re not gonna have cake anymore because you can’t make cake without either sugars or sweeteners alright so, if we get rid of both those things there’s no more cake. (pause) To me, th-the life is too short, to do away with, good things in life.”*

(DI1, P2)

*“Yeah like I don’t really mind, I would rather be a bit curvy and happy and enjoy what I eat rather than obsessively worry all the time and restrict myself of things that I want.”*

(FG6, P1)

#### 3.2.2. Angle

:: Negativity surrounding sugar, sweeteners and sweet-tasting foods.

Contrary to the positive nature of the ‘Value’ theme, the theme ‘Angle’ referred to more negative perceptions of sugars, sweeteners and sweet-tasting foods. The subtheme ‘Disinterest’ alluded to indifference. Comments suggested that some participants did not specifically include or exclude sugar, sweeteners or sweet-tasting foods in their diets, adding that concerns about sugar intake can be excessive and consuming sugar is ‘not a big deal’. Disinterest in sweeteners was related to the view that “artificial” or “synthetic” LNCS need not be deemed as worse than “natural” sweeteners.

Stronger negative feelings were grouped under the subtheme ‘Disapproval’. Comments included concerns about the short- and long-term health implications of sugar and LNCS. Sugar was perceived as non-nutritious and unnecessary in the diet, hence avoiding it was seen as a good decision, while LCNS were viewed as artificial chemicals that came from laboratories, and were potentially carcinogenic. Under this subtheme, several participants believed that sugar, LNCS and sweet-tasting foods are physically addictive, and that reducing their intake would lead to withdrawal symptoms, viewing consumption as synonymous with vices such as drug-taking.

The subtheme ‘Relativity’ contained comparative considerations. Sugar and sweeteners were viewed not in isolation, but in relation to each other, or other food components such as fat or salt. Comments suggested the concept of the ‘lesser of two evils’. For example, LNCS may be unhealthy chemicals, but at least they provide sweet taste without the detrimental health effects of sugar. On the other hand, sugar may contribute to weight gain, but at least it does not cause cancer.

Examples quotes from this theme are:

*“To me, eating sweet things, is just, quite normal! Um I-I don’t necessarily look upon it as a treat. It’s like I fancy something sweet, I’m gonna have that.”*

(FG3, P2)

*“The reason I don’t pick diet is because I heard about aspartame and I’ve heard people get tumours. It might be a myth thing but both options are bad and it’s better to do better the devil I know than I don’t.”*

(FG4, P1)

#### 3.2.3. Personal Relevance

:: To be concerned personally and/or to change one’s own behavior.

While the themes ‘Value’ and ‘Angle’ were generic in nature, many concepts were considered to affect different individuals in dissimilar ways, including the idea that some concepts were relevant to “me”, while other concepts were more relevant to other people. Many reasons were given for an individual to be personally concerned with sugar, sweeteners and sweet-tasting foods, and individuals could view these food items negatively, but did not see themselves as needing to reduce their intakes. Alternatively, some participants identified themselves as people who needed to change and thus viewed intake modifications as relevant to them. In this theme, attitudes towards sugar reduction strategies focused on specific population groups: the young, old, children, parents, pregnant women, people of lower socioeconomic status or people with obesity or diabetes. There was general consensus that the effectiveness of strategies would largely depend on the target audience and that appropriate intakes differ across individuals; hence strategies should be personalized.

Personal relevance was described in relation to a number of specific characteristics. The ‘Health and Body Image’ subtheme included references to how a person thought he or she looked in terms of body size and skin, and how healthy a person thought he or she was. Comments under the ‘Generation and Age’ subtheme reflected beliefs that taste preferences change with time and age, such that as one ages one might prefer and desire sweet-tasting foods less, rendering intake reduction irrelevant to those of older ages. Personal relevance in terms of ‘Socio-Economic Status’ referred to perceptions of economic and social class in relation to others, as a combination of income, education and occupation.

More individualized involvement and interest in consuming sugar, sweeteners and sweet-tasting foods were reflected in the subtheme ‘Stake’. Some individuals felt that they had higher tendencies than others to crave sweet or sugary foods, associated intake with socially desirable traits or saw sweet-tasting foods as staples, hence they had a greater investment when taking action towards consumption. Conversely, some participants expressed that sugar reduction was less of a priority when they were faced with a myriad of challenges, such as a heavy workload or family commitments.

An example quote from this theme is:

*“Although there are a notable amount of people now who are kinda you know driving the healthy lifestyle, there is still a lot of people who are, you know, probably more in line with where I am, and slightly beyond, which is like pffttt! Yeah, if you make it easy for me, maybe, but I’ve got other fish I need to fry right now and I’m not gonna get there.”*

(DI1, P2)

#### 3.2.4. Personal Responsibility

:: One has an active relationship with sugar, sweeteners and sweet-tasting foods.

Related to ‘Personal Relevance’, the theme ‘Personal Responsibility’ revolved around the idea that individuals have an active relationship with sugar, sweeteners and sweet-tasting foods and their consumption. Showing the greatest degree of responsibility, the subtheme ‘Informed Choice’ demonstrated complete choice over food consumption as an individual. Comments suggested that individuals valued the ability to understand and make their own decisions; they do not like to merely be told what to do, but instead want to be educated on foods, on their health implications and the rationales behind interventions. Consumption of sugar or sweeteners would depend on one’s knowledge and familiarity with each food item. Poor health was viewed as the fault of an individual for making poor decisions. Awareness and education could bring about behavioral changes, but individuals were responsible for their consumption, so there are boundaries that regulations should not cross. Some participants viewed sugar taxes as helpful in raising awareness of the high sugar content of some foods, and driving consumers to reduce intakes, suggesting even that consumers should share the cost of sugar taxes as a way of taking responsibility. However, sugar taxes were also considered unfair to consumers who keep their intakes within healthy ranges, hence any levy should be placed on overconsumption instead of consumption per se. Tactics or regulations similar to those for cigarettes such as packaging with graphic imaging, were also viewed as helpful to allow clearer identification of high-sugar products, with the recognition that this packaging could also be ignored. Furthermore, such tactics were seen as extreme and would require gradual introduction to the public. Some comments suggested that labels and guidelines, such as the traffic light rating system, were not helpful or clear enough and still require effort on the part of consumers.

An active relationship with sugar, sweeteners and sweet-tasting foods might also include ‘Self-Regulation’, a subtheme defined as managing one’s own intake of these food items. Comments suggested that some participants actively avoid or reduce sugar by rationing consumption, reducing frequency of consumption, removing any foods from the immediate environment and by preparing their own sweet-tasting foods to include less sugar. Taste preferences were also believed to be modifiable; changing preferences was a matter of habit and of getting used to new tastes. This subtheme also included the concept of balancing out one’s sugar or sweet food intake with foods or behaviors that are perceived as healthier, such as the use of sweet-tasting rewards only after intense exercise.

The idea of balance was also found in the subtheme ‘Internal Conflict’, although here the focus was more on the struggle to balance different opposing motives. Comments implied that participants struggle with motives of health versus enjoyment, resulting in the use of words such as ‘devil’, ‘naughty’, ‘demon’, ‘indulgence’ and ‘guilt’ in association with sugar and sweet-tasting food consumption. Participants expressed an “all or nothing” mentality and were unable to halt intake at times and while preferences for sugar and sweet-tasting foods were considered to be acquired and not innate, habits were also considered difficult to break. Sugar reduction was related to restraint and deprivation.

‘Motivation’ was also associated with personal responsibility. While some participants viewed themselves as responsible for their own intake, they lacked the willpower, self-care or time to change their behaviors. Other participants felt driven to make changes. Comments suggested that on top of education and modifying food products, strategies should target behaviors, such as focusing on empowerment and positive reinforcement to effect and maintain sugar reduction.

An example quote for this theme is:

*“I’ll just go ‘oh okay, that meal is mostly red for sugar [on the traffic light rating system] so I’ll make sure the other meals are not red in other areas’ so I make sure it’s like lower, a different colour for anything else I buy, and that they don’t add up. I could be buying four […] things in the red zone and be like ‘oh yeah that’s fine cause I’ve had like seven things in the orange or green’.”*

(FG4, P1)

#### 3.2.5. Understanding

:: Acquiring, comprehending and applying insights on sugar, sweeteners and sweet-tasting foods.

The theme ‘Understanding’ encompassed sub-themes associated with ‘Delivery of Information’, ‘Awareness’, ‘Perceptions’, ‘Proficiency’ and ‘Reasoning’. The subtheme ‘Delivery of Information’ focused on how information on sugar, sweeteners and sweet-tasting foods is disseminated and received. Channels included celebrities or influencers, doctors, documentaries, films, friends, social media platforms, newspapers, television programmes, the internet and hearsay and all channels were considered both reliable and unreliable. Opposing views also suggested that education and awareness was sufficient and health guidelines and promotions were aplenty, while other comments indicated that information was not widely accessible and suggested a need for education in workplaces, schools, hospitals or other organizations. Technologies such as mobile phone applications and visual cues such as labelling and advertising were seen as impactful aids in sugar reduction strategies.

The subtheme ‘Awareness’ summed knowledge and being conscious of issues related to sugar, sweeteners and sweet-tasting foods. While some participants were conscious of their intakes, were aware of concepts such as “hidden sugars” in food products and attended to the health implications and guidelines related to sugar, sweeteners and sweet-tasting foods, other participants were not mindful of these, and were unclear on the reasons behind recommendations or the origins of their opinions. When prompted specifically about the UK SDIL, some participants expressed confusion or surprise, were unaware as to whether the tax had been implemented and suggested poor awareness of the details of the scheme.

The subtheme ‘Perception’ referred to the way in which one interprets or regards information related to sugar, sweeteners and sweet-tasting foods. Perceptions included concepts of “healthy” versus “unhealthy” sugars, the suggestion that natural sugars, such as fruit sugars and honey, were healthier than artificial (chemical) sweeteners and that brown sugar was more natural and so healthier than white sugar. Participants generally viewed fruit as a healthy source of sweet taste, but suggested confusion in relation to fruit juices, fruit drinks and concentrates. The terms ‘fresh’, ‘natural’ and ‘organic’ were interpreted positively. The term “sweeteners” was generally used to refer to LNCS, and these were treated separately from “natural” sweetening agents such as honey. Low-fat or fat-free products were considered to include a high sugar content, while low-sugar or sugar-free products were considered to be high in LNCS.

‘Proficiency’ was defined as a deeper knowledge and proficiency in matters related to sugar, sweeteners and sweet-tasting foods. There were concerns that current sugar-reduction campaigns focused on sugar cubes and carbonated beverages, and hence the public may only view these items as unhealthy. There was general consensus regarding a lack of proper understanding of LNCS, and how to replace sugar with LNCS, for example, in baking or cooking. There were suggestions consequently for education on sources of sugar, sweeteners and sweet taste, and on how to prepare sweet-tasting foods with reduced sugar at home.

The subtheme ‘Reasoning’ covered how an individual applies logic while processing information in order to form inferences. Participants reported being unable to interpret the large range of available marketing, nutrition and health information, such that they were unsure whether to consume sugar or LNCS as a source of sweet taste. Responses indicated support for LNCS use for weight loss, hyperactivity, diabetes or other medical conditions. In addressing whether LNCS should replace sugar, some participants supported the use of LNCS as a short-term strategy to reduce dietary sugar, while others did not see the need for LNCS and supported sugar reduction alone. There were suggestions also that LNCS could include added health benefits such as vitamins, instead of simply providing fewer calories. With the knowledge that LNCS could provide sweet taste without the energy content of sugar, there were also concerns that guidelines may have detrimental side effects, such as reductions in physical activity when individuals switched from sugar to LNCS because they are consuming less calories, or that some strategies, such as graphic imaging on packaging, may be equally applicable to other food items, such as those high in fat or salt. There was general consensus that sugar reduction would require a holistic approach, involving government legislation, food product reformulation, education and motivation.

Example quotes for this theme are:

*“But brown rice is better for you, so surely brown sugar is.”*

(FG5, P2)

*“I think a lot has been done to educate people on sugar, but there seems to be no education on sweeteners and what they are.”*

(FG7, P4)

*“I think my concern would be if people, mis-interpreted the message that said sweeteners are okay, and sugars are less okay. People might think, well I won’t bother exercising now and they think then if if I just turn to sweeteners.”*

(FG3, P1)

#### 3.2.6. It Is Not Up to Me

:: One takes a passive approach towards sugar, sweeteners and sweet-tasting foods, because intake is subject to other factors.

Contrary to the active involvement of the individual in the above three themes, the theme ‘It’s not up to me’ detailed a passive approach to sugar, sweetener and sweet food consumption. Food intake was considered to be ‘Beyond Individual Control’, to be determined instead by ‘Strategies and Regulations’ or ‘Deception’. The subtheme ‘Beyond Individual Control’ referred to factors that individuals felt unable to control. Food choice and intake were considered to be determined by the availability and accessibility of sugar, sweeteners and sweet-tasting foods and the social and cultural environment, including family, friends and peers. Some participants suggested that the normalization of obesity could increase sugar consumption, as high sugar intake was seen as acceptable or typical, while others suggested that society is becoming healthier and that lower sugar consumption is more acceptable. The sugar content of foods was seen as unnecessarily high, a fault of food manufacturers, and advertising and marketing strategies were considered to be aggressive. Sugar reduction strategies were also considered to compete with these influences. One reason that was given for failing to notice the SDIL, for example, was that large price fluctuations in the economy may mask small tax-related increases in prices. For some participants, sugar reduction was also beyond one’s control because the addiction to sugar was difficult to overcome and would require professional help.

The subtheme ‘Strategies and Regulations’ referred to official legislation and large-scale measures. Comments implied that healthy food consumption was the responsibility of the government. Sugar taxes and the promotion of reduced-sugar foods as default options were viewed as beneficial for driving manufacturers to lower the sugar content of foods, but there were concerns that implementation was dependent on individual manufacturers, and that the food industry could choose to reject measures such as sugar labelling or graphic imaging. Current government dietary recommendations, such as keeping below 30 g of free sugars per day, were also seen as possibly unrealistic.

Finally, the subtheme ‘Deception’ incorporated ideas of traps and tricks used by food manufacturers and distrust of the food industry. The food industry was considered to be corrupt, to intentionally load sugar or sweeteners into foods, and to mislead consumers with unclear labels; there were suggestions that consumers were pitted against food manufacturers and the tactics of the latter would triumph. Comments also reflected distrust of current food labels and of the information produced by health professionals and scientific researchers.

Example quotes for this theme are:

*“I think people need professional help! You know for sugar? Cause of the fact that I’ve I- I- y-yeah. I think she’s right. It is a drug (pause) and when I when I need, I need it.”*

(FG2, P6)

*“But, at the end of the day, you know, if that doesn’t work, is like people are children you know, [authorities have] to tell them off and the only way is punishment! Isn’t it?”*

(I1, P1)

*“I think it’s sneaky how much they put in stuff, it can be hard to stick to your plan or keep things in moderation when companies load things with sugar and fat.”*

(FG6, P3)

## 4. Discussion

This work aimed to explore attitudes towards sugar, sweeteners and sweet-tasting foods in a sample of the general public of the UK. A combination of seven focus groups, two dyadic and one solo interview was conducted to elicit a wide range of attitudes. Attitudes were grouped into six main themes: ‘Value’, ‘Angle’, ‘Personal Relevance’, ‘Personal Responsibility’, ‘Understanding’ and ‘It’s Not Up to Me’. Both positive and negative attitudes towards sugar, sweeteners and sweet-tasting foods were expressed across these six themes, largely dependent on the individual.

Attitudes captured by the theme ‘Value’ demonstrated a range of advantages to using and consuming sugar, LNCS and sweet-tasting foods, from food preservation, energy provision and reduction, to pleasure and enjoyment, while less positive attitudes were grouped into the theme ‘Angle’. Comments in this theme demonstrated attitudes that were negative, uninterested or dependent on competing alternatives. Perceived benefits are common reasons for consuming foods [42,43], and with the inherently rewarding nature of sugar and sweet taste [21], benefits such as taste and pleasure are commonly reported as reasons for consuming sweet-tasting foods [12,13,14,15,16,20], alongside reasons associated with emotions [44,45,46] and memories [46,47]. Perceptions of health benefits are also frequently reported in association with the consumption of both sugar and LNCS [12,16,17,18,19,29,30,31], as are negative attitudes and health concerns [13,14,16,22,31].

Compared to the straightforward nature of the themes ‘Value’ and ‘Angle’, the theme ‘Personal Relevance’ focused on more complex ideas that some people may be or may need to be more concerned with sugar, sweeteners or sweet-tasting food consumption than others. Differences between individuals were recognized based on demographic characteristics such as gender, age and socioeconomic status, on personal interests such as the importance of health or appearance to each individual, or personal situation. The importance of personal relevance for influencing dietary behaviors has been previously detailed elsewhere [48,49,50,51]. Recognition of varied motivations across individuals is also paramount in this literature [48,49,50,51]. The existence of contrasting concerns and barriers toward healthy food consumption among differing population groups is also clear in earlier work on sugar, sweeteners and sweet foods [13,14,15,16,17,30,31].

The ideas of individual differences and individual motivations were also noticeable in the theme ‘Personal Responsibility’. This theme centered around the idea that one has an active relationship with sugar, sweeteners and sweet-tasting foods. Participants liked to be fully informed over the advantages and disadvantages of food ingredients, such that they can make their own choices, and regulate their intake for themselves. This idea of personal choice and personal control is apparent elsewhere in the literature relating to sugar and LNCS use [14,17,31], and is found in the literature relating to food choice, and particularly the acceptance of novel foods, diets and dietary guidelines [52,53,54]. Increased perceptions of personal choice, control and responsibility have also been linked with more healthy dietary consumption and a healthier body weight [48,55,56,57]. Some participants recognized this self-regulation as resulting in inner conflict or requiring additional motivation, but the over-riding idea of personal choice and responsibility remained paramount.

Linked to ideas of personal choice, and the need for information to enable this, the theme ‘Understanding’ specifically considered the means by which consumers gain and process information. Subthemes recognized the importance of both conventional and novel types of information and information channels, the value of awareness for informing and changing intakes, the importance of diverse perceptions and the use of wide-ranging and assorted justifications to rationalize sugar, sweetener and sweet food intakes. Information is repeatedly requested in response to dietary challenges [12,14,15,16,17,18,19,20,23,30], and is often available e.g., [25,26], but inaccessibility by certain individuals or population groups, misunderstanding and confusion often still remain [13,14,15,17,18,19,23,30,31].

Contrary to the active engagement in themes ‘Personal Responsibility’ and ‘Understanding’, the theme ‘It’s not up to me’ allowed individuals to take a passive approach towards sugar, sweeteners and sweet-tasting foods, and recognized that intake is subject to other factors. The influence of external influences, such as the food supply and the social and cultural environment, in dietary intakes is well recognized [11,12,13,15,16,17,18,19,20]. Subthemes within this theme also gave the responsibility for sugar and sweetener consumption to governments and the food industry, and permitted an even further reduced role for the individual through ideas of ‘deception’. Previous research has also revealed this blame towards external organizations [12,13,15,17,18,19,23,57], and this distrust in health professionals, government agencies, and in the food industry [13,16,23,58,59].

Clear attitudes towards policies and strategies for reduction were also expressed, and attitudes within each of the themes had clear implications for reducing sugar intakes. Clear benefits and disadvantages of sugar, sweeteners and sweet-tasting foods, plus the importance of personal relevance suggest a need for individualized and personalized strategies. The active engagement demonstrated under the themes of ‘Personal Responsibility’ and ‘Understanding’ suggests a need for greater awareness, education and the many independent ideas in the theme ‘Understanding’ suggest the potential need for a range of distinct types of information or education for instigating dietary change. The media portrayal of scientific publications and policy discussions has been shown to influence consumer perceptions of sugars and sweeteners [60].

The need for personal relevance, and the possible differing attitudes based on population group, however also suggest that information may be more acceptable and more effective when tailored to specific perceptions and motivations. Similar concerns over acceptability, use and effect have also been suggested in the literature on sugar, sweeteners and sweet foods [12,13,14,16,19], and work on other aspects of nutritional information also highlights the need for different types of information for individuals [60,61]. Information alone, however, may not to be enough [12,18], and within the theme ‘Personal Responsibility’, strategies for change also focused on empowering the consumer; providing the information to allow individuals to take responsibility for their own consumption and motivating consumers to act for themselves. Clear opposing strategies for change were also apparent within the theme ‘It’s Not Up to Me’. Here, strategies for change relied entirely on government legislation and regulation of the food industry, but possible backlash as a result of restrictions and deception were also apparent. As detailed by some participants, reduced intake of one food-type or ingredient also requires a workable alternative. Promotion of the value of alternative foods, and the benefits of these alternative foods, may add weight, particularly if these benefits are targeted to different consumer groups with their own value priorities.

The need for ‘Personal Relevance’ may make it hard to make changes to intakes on a population-wide basis. An absence of evidence for the prevalence of different attitudes and distinct consumer groups, furthermore, makes prioritization of certain approaches or strategies difficult. Evidence of the dominant attitudes in those in greatest need of change would be of value. Arguably, those in greatest need of reduced sugar intakes are those who are consuming large amounts of sugar, and those who are likely to be differentially affected by these intakes—those already experiencing or likely to experience related health conditions, such as dental caries, cardiovascular disease, Type II diabetes, overweight and obesity [1]. Future work should investigate the prevalence of the attitudes identified in this study in a large representative sample of the UK population, investigate the dominant attitudes in specific individuals, and the relationships between specific attitudes and dietary free sugar intakes.

The study was limited by its qualitative nature, allowing inclusion of a limited number of participants, however, recruitment was undertaken across a range of venues and minimal inclusion criteria were used to gain a range of individuals with a range of attitudes. Bias in the participants who volunteered and participated may have led to the demonstration of certain attitudes more than others, but we think it is unlikely that any important concepts have been missed. Discussions were also contextual and will reflect the prevalent attitudes and sugar-related policies in the UK at the time the study was undertaken. New legislations on advertisements and the labeling of foods high in sugar may shape consumer attitudes in the future [62]. The analyses may also have been affected by the backgrounds of the researchers undertaking this work. Attitudes were elicited using a standardized moderator guide, and all focus groups were undertaken with the aim of eliciting as many attitudes as possible.

## 5. Conclusions

This study identified six themes to describe attitudes to sugar, sweeteners and sweet-tasting foods in a sample of UK consumers: ‘Value’, ‘Angle’, ‘Personal Relevance’, ‘Personal Responsibility’, ‘Understanding’ and ‘It’s Not Up to Me’. Individuals held indifferent, positive or negative perceptions of sugar, sweeteners and sweet-tasting foods, which could be influenced by their perceived competing alternatives. Individuals also reported varied engagement with intakes of these foods, taking either an active or passive approach to modifying their intakes. Potential strategies for reducing free sugar intakes were reported, but differing perceptions of likely value were also suggested. The existence of individual differences in perceptions, motivations and priorities may indicate a benefit for differing approaches. For the greatest population benefit, evidence of the dominant attitudes in those of greatest need of reduced free sugar intakes would be of value. Future work will investigate attitudes in a large representative sample of UK consumers, the dominant attitudes in specific population groups, and investigate the associations between these attitudes and free sugar intakes.

## Data Availability

The data presented in this study are available on request from the corresponding author. The data are not publicly available due to privacy of audio transcripts.

## Supplementary Materials

The following are available online at https://www.mdpi.com/article/10.3390/foods10061172/s1; Figure S1: Free sugars intake recommendation by Public Health England, and sugar contents in popular drinks, all portrayed with number of sugar cubes; Figure S2: Various sources of sweet taste; Figure S3: Examples of what packaging can look like on products containing sugars, with graphic imaging similar to that that has been used for cigarettes under The Standardised Packaging of Tobacco Products Regulations 2015; Figure S4: An actual BBC News article on the SDIL, illustrated as a newspaper clipping; Table S1: Definitions and quotes for each theme and subtheme; Table S2: Moderator Guide.
