# Supplementary material for: Sweet Talk: A Qualitative Study Exploring Attitudes towards Sugar, Sweeteners and Sweet-Tasting Foods in the United Kingdom
Source: Foods. 2021 May 24;10(6):1172. doi: 10.3390/foods10061172 (PMC8225159; doi:10.3390/foods10061172)
Supplement: Supplementary file 1 [file foods-10-01172-s001.zip › foods-1176262-supplementary.pdf]

Supplementary Materials

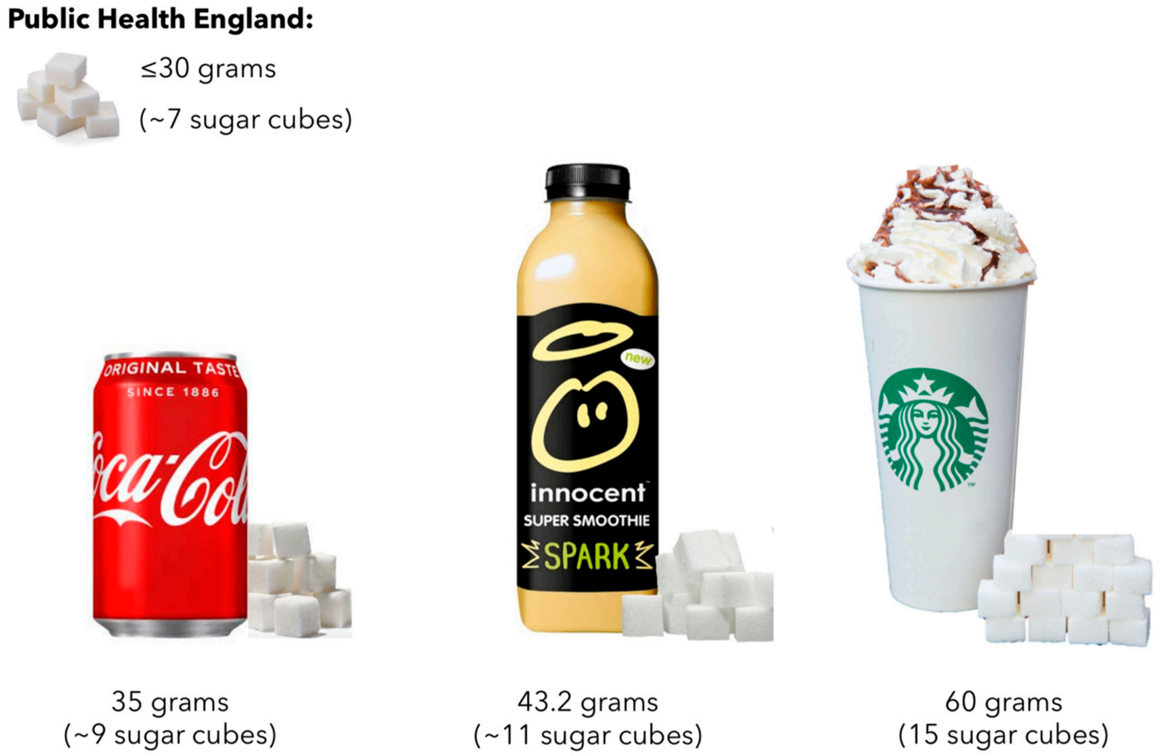

Figure S1. Free sugars intake recommendation by Public Health England, and sugar contents in popular drinks, all portrayed with number of sugar cubes.

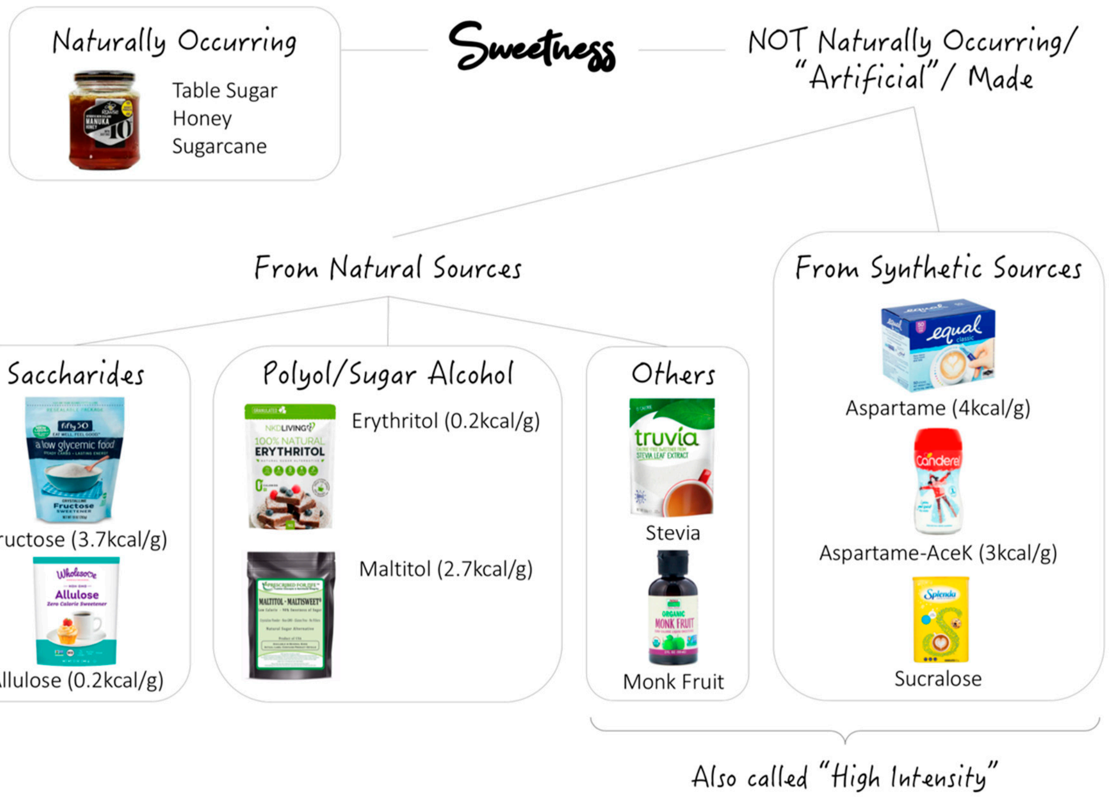

Figure S2. Various sources of sweet taste.

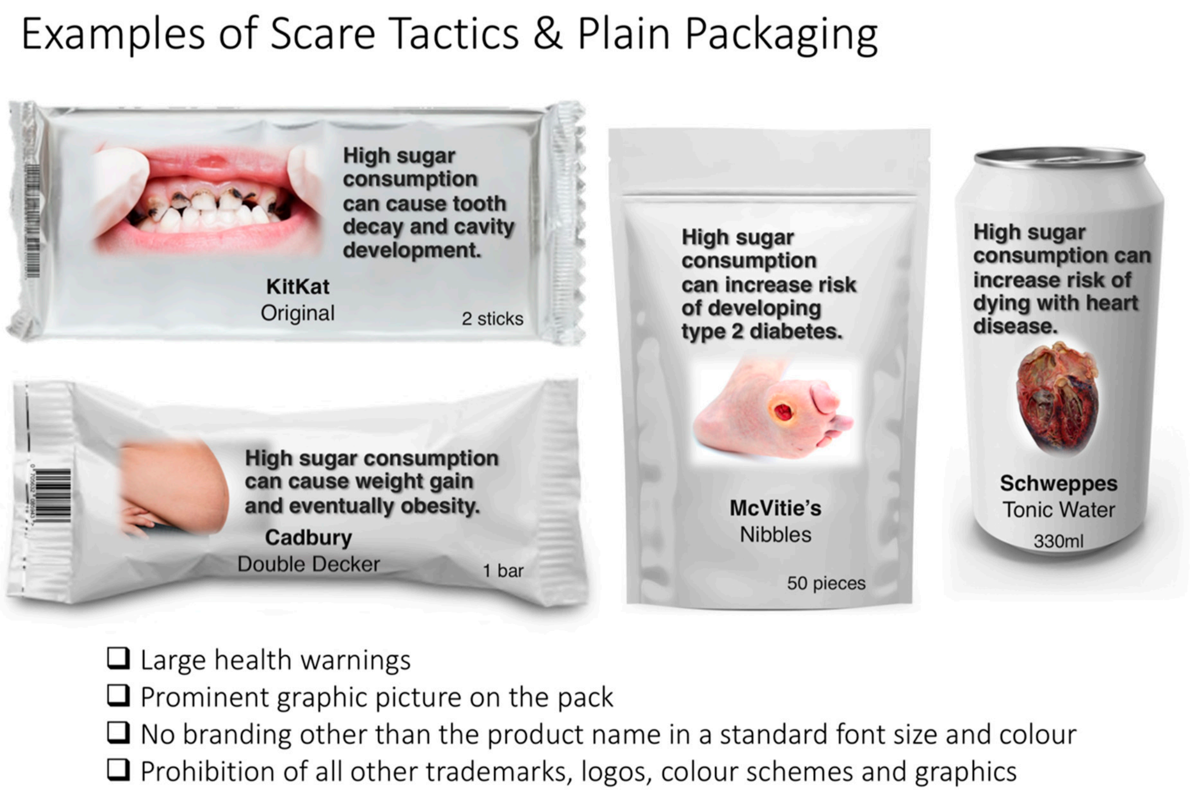

Figure S3. Examples of what packaging can look like on products containing sugars, with graphic imaging similar to that that has been used for cigarettes under The Standardised Packaging of Tobacco Products Regulations 2015.

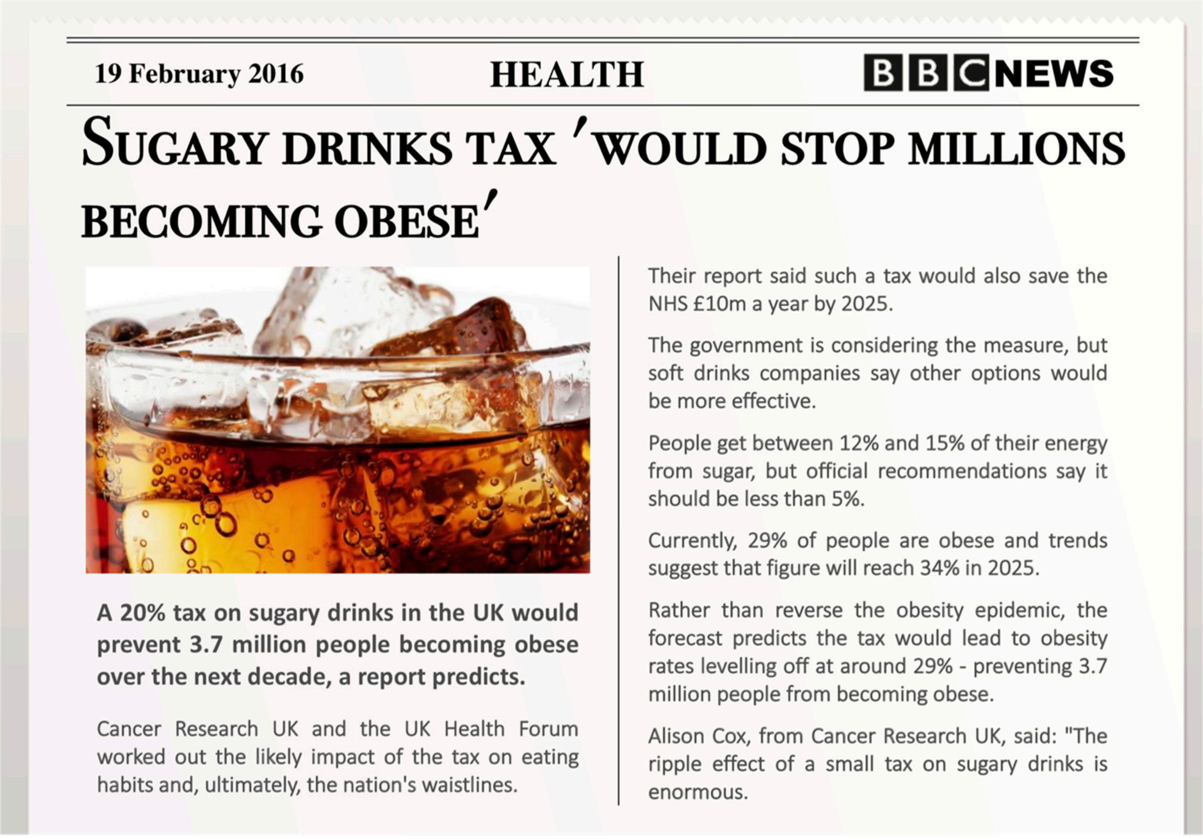

19 February 2016 HEALTH BBC NEWS

SUGARY DRINKS TAX 'WOULD STOP MILLIONS BECOMING OBESE'

A 20% tax on sugary drinks in the UK would prevent 3.7 million people becoming obese over the next decade, a report predicts.

Cancer Research UK and the UK Health Forum worked out the likely impact of the tax on eating habits and, ultimately, the nation's waistlines.

Their report said such a tax would also save the NHS £10m a year by 2025.

The government is considering the measure, but soft drinks companies say other options would be more effective.

People get between 12% and 15% of their energy from sugar, but official recommendations say it should be less than 5%.

Currently, 29% of people are obese and trends suggest that figure will reach 34% in 2025.

Rather than reverse the obesity epidemic, the forecast predicts the tax would lead to obesity rates levelling off at around 29% - preventing 3.7 million people from becoming obese.

Alison Cox, from Cancer Research UK, said: "The ripple effect of a small tax on sugary drinks is enormous.

Figure S4. An actual BBC News article on the SDIL, illustrated as a newspaper clipping.

Table S1. Definitions and quotes for each theme and sub-theme.

Value | What sugar, sweeteners and sweet-tasting foods can provide.		
Taste	The flavour of sugar, sweeteners or sweet-tasting foods	"It still has to taste good I think that's the thing." (DI1, P2)<br>"Does the natural, yes I definitely consider them? Um, it obviously comes down to taste? Um I probably want and, get it and make put it in my own recipes and see how it (pause) changes things from having sugar in them?" (FG3, P3)<br>"And the taste of soda is better when is with sugar and if is (pause) if there's no sugar, then is isn't worth drinking it." (FG2, P3)<br>"The taste, sweeteners are disgusting (laughs) in my opinion they taste very different and I, I don't like it." (I1, P1)<br>"You wouldn't think that would be drinkable! Would you. You'd think it would just be so over sweet!" (FG7, P4)<br>"I just don't like sugar in my coffee but that's about it. I do eat sugar all the time, so (laughs)." (FG2, P6)
Pleasure	The enjoyment and satisfaction derived from experience with sugar, sweeteners or sweet-tasting foods	"The fact that people just enjoy th- their drink, with sugar. Is just that is something that you cannot change because some people most people, to be fair, don't even care about the (pause) Health impact." (FG2, P6)<br>"They just, just lookin' at them they come in a lil plastic box. Just don't like the look of it I just don't like the idea of it? Um (pause) yeah I don't use sugars, um in my coffee anyway I and I don't cook so, um (pause) yeah I don't really have a sugar in my in my house. But um, if I had to, I'd go for sugar not a sweetener I just don't like the look of them I don't know. Just look like little pills (laughs) something puts me off." (FG1, P4)<br>"Let's say we're not gonna have cake anymore because you can't make cake without either sugars or sweeteners alright so, if we get rid of both those things there's no more cake. (pause) To me, th-the life is too short, to do away with, good things in life." (DI1, P2)<br>"When you love something, you can-not let go, right? (laughs) You will make excep-exceptions for the thing that you really love." (FG2, P3)<br>"I always go full fat, full sugar. You know if, again is about taking responsibility. If you decide to put something in your mouths, you want to have an experience of it. It's it's fine, with it. Just take th-the real thing as opposed to artificial things and, just eat less of it and just appreciate what you have!" (I1, P1)

		"Although I do like a Jager bomb… Yeah so if I'm going out then it's a bit different, but all my inhibitions go out the window and I just let loose if I'm drinking." (FG6, P2)
Special	Not an everyday affair with sugar or sweet-tasting foods	"That taste of Cadbury's chocolate? Um… it it just relaxing. It feels like a treat." (FG3, P3)<br>"But for me I would rather not buy diet or reduced sugar things I would make the sugary things more of a treat and have less often." (FG7, P3)<br>"So I never really (pause) m-my mom's always been sort of health, conscious so we (pause) it's probably bad on a Sunday night we used stop off at this local shop, and just get so much. So many sweets and (pause) and, bad things but. Yeah and just our day to, to have that and that was after we'd, been on, been round to my grandparent's for Sunday dinner so it was, sort of like our, cheat day y'know? But (pause) yeah I don't do that, too often." (FG1, P1)<br>"Yeah about it being novelty? When coke you know and all those, sugary drinks. Yeah. When they became novelty and everybody wanted, to have it type of thing and, it wasn't so readily available before so everybody jumped on the bandwagon really." (FG1, P4)
Emotion	Sentiments and feelings derived from sugar or sweet-tasting foods; influence of mood on intake	"For me I think happy uh like this sounds weird. But like happy thoughts? I'm like, pudding is a good thing." (DI1, P2)<br>"I think that in the psychology aspect? Uh some people looking for sweetness when they are sad or depressed." (FG2, P2)<br>"It makes me happy and that's all I care about so (laughs)" (FG2, P6)<br>"Yeah like I don't really mind, I would rather be a bit curvy and happy and enjoy what I eat rather than obsessively worry all the time and restrict myself of things that I want." (FG6, P1)<br>"When I drink juice? I always feel much more healthier." (FG2, P4)<br>"It depends yeah, depends on the mood." (FG4, P4)
Worth	Weighing of cost and benefit, for sugar, sweeteners or sweet-tasting foods	"And they had this coffee cake or whatever (pause) on fifty percent off. So I thought I'll have that. And uh, yeah of price. Price. And it was a pretty big slab." (FG1, P2)<br>"I maybe care about the price! Rather than discriminate between sugar and sweetener." (FG3, P1)

		"it's to understand what the health benefits if there are any or there are, and I assume there'll be a cost impact as well? And then it's not like we're running out of sugar? (laughs) So, it's not gonna help the planet. So I need to an angle to really think why why would I want to, change and if (pause) if I saw in, products that are on sale? (pause) I would look it up first and think okay, again why? And then I'll think about it." (FG3, P3)<br>"Yeah I associate it to weight loss and it's better when I'm counting calories." (FG6, P3)<br>"Let's say you need uh, a a boost for, the energy boost you can get but you can get it in other ways but (pause) uh sort of nutritionally as far as I understand there's not much benefit to (pause) unlike sh- salt, where people need, a little bit of salt in their diet." (FG1, P2)
Angle | Negativity surrounding sugar, sweeteners and sweet-tasting foods.		
Disinterest	Indifference towards negativity surrounding sugar, sweeteners or sweet-tasting foods	"Yeah, balanced rather than just the sugar or just the fat but you know, in order to keep ourselves healthy. We need to do this. we need to have that. we need to sleep. We- a package rather than (pause) sugar." (FG3, P1)<br>"Like I don't think it's necessary in this case but I mean, personally, yeah I-I'd I have some biscuits, I'd chocolate here and there, and (pause) um but there are things so (pause) there are- so what I find myself, doing (pause) is, a chocolate bar is a chocolate bar and, you can get one without sugar if you want, but I don't go around looking for that particularly. (pause) Uh so when I have one I have one." (FG1, P2)<br>"I think people do have do have sugar just because they like it but if they didn't have to have it, they wouldn't, have sort of withdrawal symptoms and, and what not, from, from not having sugar." (FG1, P1)<br>"I don't think that it's necessarily is uh (pause) I don't think it's necessarily the case that because something we can label it and call it uh because it is and we call it artificial or synthetic, it is necessa- that necessarily means, that it is (pause) less uh or, it's not as good or it is worse than something natural." (FG1, P2)<br>"To me, eating sweet things, is just, quite normal! Um I-I don't necessarily look upon it as a treat. It's like I fancy something sweet, I'm gonna have that." (FG3, P2)<br>"N-nothing really comes, comes to my mind (pause) cause I'm not always thinking when's my next, cake coming or something (pause) so it's not, really I don't really have a (pause) it doesn't mean I don't enjoy it sometimes when I have it but." (FG1, P2)

Disfavour	Serious and strong negativity or disapproval towards negativity surrounding sugar, sweeteners or sweet-tasting foods	"Yeah I think we mainly covered the (pause) um, so for example, both the sugar, table sugar and artificial sweetener, they're both artificial." (FG2, P5)<br>"I think I think people need professional help! You know for sugar? Cause of the fact that I've I- I- y-yeah. I think she's right. It is a drug (pause) and when I when I need, I need it. It's not like (pause) uh, oh I should have a cake, oh I shouldn't have. It's more like, I NEED A CAKE… So yeah I feel I feel (inaudible) education, but I think people are not really aware of the fact that it is quite addictive? And it it is quite, um harmful to your body? It is important but also professional help, might, yeah. Help people." (FG2, P6)<br>"Sugar's a highly addictive substance." (FG1, P2)<br>"And that's the one that, a couple of years had a lot of bad press. Alright and it was like, y'know people were saying oh it can cause (pause) illness like serious illness something as even like people were saying like… It can cause, cancer and all those kinda stuff." (DI1, P2)<br>"It's delivered, to refineries in, in tankers with the skull and crossbones, symbol on it… Because of the chemical contents within it." (FG3, P2)<br>"Yeah I think there's chemicals in them um (pause) same what they put in diet, um things so I just stay away from it, d- yeah it puts me off." (FG1, P4)<br>"You're just basically fooling your brain into like, your body's like having sugar but it's not actually? So it's actually making everything worse? (pause) So that's what I heard." (FG2, P6)<br>"Yeah because you kinda get addicted as well to the sweet taste you know? Um, I think if you are on a, low-sugar diet for a- awhile, even for a week and I've heard that from, from people you know once they go back, and go back to their normal diet, everything taste so much sweeter and I think it's just, our brain kind of get ad- not addicted but used to it." (I1, P1)<br>"They're consuming things, and probably getting hooked onto a taste (pause) without really realising it. Um and then it's difficult for them to shake that off as they get older." (FG3, P2)

Relativity	Whether sugar, sweeteners or sweet-tasting foods are good or not, is relative to what you are comparing it with	"The reason I don't pick diet is because I heard about aspartame and I've heard people get tumours. It might be a myth thing but both options are bad and it's better to do better the devil I know than I don't." (FG4, P1)<br>"Um, the fact that it's more naturally processed. So I would, go, on sugar rather than sweeteners just because it's, it's less processed really. (pause) So you know, the less, th-th-the less processed it is the better. I think it is, for me." (I1, P1)<br>"Yeah I go for normal as well, I really hate the taste of diet stuff and I think it's more natural even though it's probably still not good for you." (FG4, P4)<br>"Advantages? As in you're getting the sweetness without the sugar." (FG5, P1)<br>"It's just if you'd like the sweet taste. Like my dad for example, has sweeteners in his tea and coffee, as he couldn't have a coffee without anything in it. He chooses a sweetener over sugar as he feels it is healthier for him. (pause) Oh I guess if you have got for example younger children or even yourself who massively react to sugar and you notice a change in their personality then maybe sweeteners would be better in that situation." (FG7, P4)
Personal Relevance | To be concerned personally and/or to change one's own behaviour.		
Health and Body Image	How a person thinks he/she looks in terms of body size and skin; how healthy a person thinks he/she is	"I am eating way too much sugar. but I'm also doing way more exercise than the average person (pause) I would say average. Um (pause) so is there actually a link? Am I actually safe to eat the amount of sugar I'm eating or is there actually a problem and I shouldn't be? I don't know the answer to that but because, I'm not having any problem as you were saying as well. Then you know, th-there's nothing to spur you to change..." (FG3, P3)<br>"I think it depends on, on the individual! Because I do think they-they have theor (pause) advantages if, if you take someone that is (pause) morbidly obese (pause) would you be more concerned about the chemicals that you're puttin' in their body? Or if the sole aim for them would the most important thing factor was them to lose weight, (pause) then perhaps uh (pause) a sugar alternative might be the, the best solution in that (pause) in that case? If they were tryin' to lose weight, because they were at risk of, some kind of serious heart disease or something?" (FG1, P1)<br>"There's a lot of messages around and I think now, there's more of an emphasis on (pause) people (pause) lookin' at it thinking right what do I do? But th-then some people think that'll never happen to me. Whoosh! Shall eat what I want." (FG3, P1)

		"Nowadays (pause) well I am aware of (pause) like sugar-related issues health issues. Um but I don't think I eat so much sugar that I should (pause) cut it down? Uh I would say that nowadays, my (sigh) food, um (pause) i-if I was to change, anything, it would be (pause) related to fat rather than sugar? (pause) I think?" (FG1, P3)
Generation and Age	Whether a person identifies with being a child, a youth, an adult, or an elderly; belonging to an "younger" or "older" generation	"I do, for the children? (Laughs) [But, not (laughs) but not for ourselves!] Not for ourselves yeah." (DI1, P1 and P2)<br>"Um, so I think also (pause) embeddin' and implementin' education that type of education into schools would be really, helpful cause, back then, there was nothing, nothing like that, say you don't even think about the implication of it actually w-what you doin', to your body? Um, but (pause) you know, as I got older, my you know, my -my taste changed and I don't crave sweets as much." (FG1, P4)<br>"I do think it's um, (pause) needed in workplaces cause I think you've got a generation (pause) perhaps slightly older than us? So it may be into you- into your forties and fifties. Who, haven't had any awareness of any of this, haven't been really affected by kinda social media drives because they are not really a social media generation. So I think there is a, a generation above us, that is kind of like, I don't know kinda hang on, we-we've missed them and actually you could capture them with some workplace intervention." (DI1, P2)<br>"For elder people I don't know like maybe, only for label, it's good for them because they know already, at their age. Because of low sugar is better for them. (pause) But for like um (pause) young age, have to like educate more. (pause) Yeah. Give information?" (FG2, P1)<br>"But it isn't that bad for you, and children's brains are still developing but older bodies are not as affected by these chemicals but for young children to be drinking aspartame every day." (FG5, P1)
Socio-Economic Status	The measure of a person's economic and social class in relation to others; a combination of his/her income, education and occupation	"I'm sure someone somewhere has done this. (pause) But if you were to say okay who are the, leading consumers of foods and drinks that are high in sugar? I'm guessing we would find that it's the people that there are. Have less money." (DI1, P2)<br>"I don't mean to sound judgmental but the people I see who usually consume high energy and high sugar drinks are usually people who look quite rough and poor." (FG6, P3)<br>"It's got to be a holistic approach, because we are sat here as essentially comfortable financial group, but I think you have to educate the masses and peoples dietary budgets are very different. The problem is you can buy a burger king for two pounds against buying fresh fruit and vegetables which are much more expensive. Unfortunately, people are always going to default to the cheaper, easier option. Changes need to be made at the

		legislation level, hitting the source, who make it so readily available. We need to put more emphasis on the suppliers so that they take responsibility for it." (FG7, P2)
Stake	The involvement and interest in the process and outcome, of consuming or reducing sugar, sweeteners or sweet-tasting foods	"You know, cause [participant name] sees it as a, a treat. Whereas I will kinda see it more as like a staple, like something you have every day. Yeah or, or at least more often than not. Maybe not every day. But more often than not." (DI1, P2)<br>"Although there are a notable amount of people now who are kinda you know driving the healthy lifestyle, there is still a lot of people who are, you know, probably more in line with where I am, and slightly beyond, which is like pfftttt! Yeah, if you make it easy for me maybe but I've got other fish I need to fry right now and I'm not gonna get there." (DI1, P2)<br>"I don't use them. I mean (pause) since I don't add stuff anyway, I'm not um, don't add sugar or artificial sweetener." (FG1, P2)<br>"No I don't have any problem like cause I don't have cravings for it so I'm, I'm good." (I1, P1)<br>"I don't add sugar really to anything. (pause) and um, I, for me personally I wouldn't ever stop to think about whether a sweetener was good bad or indifferent I just don't use them so they're not really on my radar." (FG3, P1)<br>"I feel like all of the things I prioritise in my shopping list low sugar isn't really (pause) something I would think of." (FG5, P2)<br>"And maybe relating it to their experience for example if you're talking with people who are, very much into putting sugar in their coffee. You (pause) kind of shape your, education, based on that. Because they are interested in that one. If you talk about something else and if they don't already consume it they won't listen but if you relate it to their, experience they will listen." (FG2, P5)
Personal Responsibility | One has an active relationship with sugar, sweeteners and sweet-tasting foods.		
Informed Choice	The ability to understand each option and make the decision	"I don't think it works telling people these days, don't do this. (pause) Because you know, if someone said to me don't do that, I'd just gonna ignore you and do it. (pause) If someone said hey, I know you like that and that's fine. But just so you know, this is a better option. And this is better for you and here is why. But I you know what, I'd listen to that." (DI1, P2)

		"But until such point is I know, what are the sweeteners I can use and how they work versus sugar, I would still carry on with sugar until I have that knowledge." (DI1, P2)<br><br>"It's so hard to identify these days what is really, bad for you what is not so bad because you have to go and read it all. You know um whether that would be a easier way to do it maybe that, would it be a step too far, could we do something in in between? But I think it would be beneficial to help consumers identify more easily (pause) what what's in food and (pause) what's bad for you." (FG1, P4)<br><br>"There's a lot of (pause) questions about (pause) uh how far (pause) the difference between providing information about what people might want to do and actually trying to get them to do something else (pause) now I find that it's actually intervening in their, in their own private life, for example." (FG1, P2)<br><br>"I think you have to be s-sort of consciously aware of the quantities and the (pause) macronutrients that's (pause) in a product. Um (pause) when you're readin' reading those labels and and trying to make a sort of, an informed decision on on what you then buy I think that I think that's one of the real issues?" (FG1, P1)<br><br>"No I don't think so, but because I'm I don't know what's in it and I've never kind of I always never curious cause I always knew what's in the cube so I always use the cube. I guess it's it's kind of what you know about it and familiarity." (FG2, P5)<br><br>"Because at the end of the day, its' a free world and and we cou we're all at liberty to make our own choices. But (pause) the information just needs to be. A bit clearer." (FG3, P2)<br><br>"Yeah like it's education isn't it? But they can't just put that out there as, we think you should eat less without explaining why and who it affects and what." (FG3, P3)<br><br>"I don't think that's (pause) that's gonna be an answer to take something and replacing with something else. So, as I said we don't know the effect long term of all those artificial sweeteners in our bodies as well! So why would you do that?" (I1, P1)<br><br>"I think a combination would work well, like education and scare tactics would work together, because people would understand the reasons behind it rather than just being told 'don't do this'." (FG6, P3)<br><br>"Yeah but at the end of the day if the customer wants it then surely they should pay for it? They aren't forcing them to consume it." (FG6, P1)

		"There should definitely be traffic light thing, it's good cause it's a rule and you can see it on everything and it's consistent over all foods, education need to be a simple rule so they can work out in the shop like you say you're not going to get your phone out if you're in a hurry." (FG5, P4)
Self-Regulation	Managing one's own consumption; modifying own behaviour such as intake or exercise accordingly	If I, yeah doing sport and I think oh! I can afford to, have a can of Fanta because. You know? I've just done three hours on the court!" (FG3, P3)<br>"Um, if I've got something sweet in the house (pause) I'll eat it and that's possibly why I don't buy, that sort of thing from (pause) from the supermar- if I want something sweet, I'll get it." (FG1, P1)<br>"The traffic light system, occasionally I'll, if I think I'll, all I'll measure it by is like adding up what's red in the sugar zone, I'll just go 'oh okay, that meal is mostly red for sugar so I'll make sure the other meals are not red in other areas' so I make sure it's like lower, a different colour for anything else I buy, and that they don't add up. I could be buying four fucking things in the red zone and be like 'oh yeah that's fine cause I've had like seven things in the orange or green'." (FG4, P1)<br>"Well I buy basic cornflakes and cut up a banana really thinly and I never eat raisins cause they're unappealing I do that now cause I know I've got to eat more fibre and fruit helps it carry it through I have chocolate one or two times a week, not a big bar, it's going on average like I binge and then I don't touch anything for two weeks." (FG4, P1)<br>"I really like home cooking so I don't like to buy things cause actually when you look at the content of sugar say in a, pre-made Bolognese sauce or something, it's really really high. Um (pause) and I uh (pause) so I-I like to like, just cook things like make them, from the ingredients, rather than using a tin of food or whatever or a bottle of food." (DI1, P1)<br>"And I get them in bulk from those other things that I shouldn't have too much of? I kinda take them out of (pause) th- the healthier options." (FG3, P3)<br>"When um this meal I drink water, next meal I will like, treat myself like have a cup of (pause) um soft drink? Yeah. (FG2, P1)<br>"I try and balance it, some days I think I have way over two thousand but the next day I'll be healthier and have a salad or something." (FG6, P1)<br>"And if I need to have one KitKat, I will have that KitKat because. It won't affect me that much. Cause it's just once a month or something." (FG2, P5)

		"I programme myself to like it too I think I'm used to it, a lot of people say they can't taste it but I don't really care, you get used to bitter tastes, like I've got used to black tea, I used to hate it and now I love it." (FG5, P1)<br><br>"To be fair I started using honey instead of sugar in tea and coffee so I started doing that and it doesn't even taste that different. I bought sweetener for the first time yesterday, Canderal." (FG5, P2)
Internal Conflict	Struggling to balance out motives, feelings and behaviours; pull and tug between two opposing voices	"Whether I'll ever completely get away from it? I don't know. But I think I'd probably move towards, kinda the way that [participant name] has Innocent smoothies which is, you have them every so often, because it's nice. But it's not part of your staple day to day. Um, so I think it'd probably be more towards that I can't ever act- can't see myself ever completely ditching it?" (DI1, P2)<br><br>"I honestly don't know because I do eat a lot of sugar (laughs) So, uh removing from my life I think that would be very very challenging for me." (FG2, P6)<br><br>"Some of the time that will be a fact that people (pause) are gonna consider when going to cause if you say oh it's the same you think, do I want to be healthy? Or do I not? But I, don't know what that is, thirty pence cheaper?" (FG1, P1)<br><br>"Let's say we're not gonna have cake anymore because you can't make cake without either sugars or sweeteners alright so, if we get rid of both those things there's no more cake. (pause) To me, th-the life is too short, to do away with, good things in life." (DI1, P2)<br><br>"Yeah or a bit of fruit but I don't always want a bit of fruit, I don't want that sort of sweet I want a bit of naughty sweet." (FG5, P3)<br><br>"You feel you being demonised cause actually you deserve that treat cause you've worked for it." (FG3, P3)<br><br>"I do crave fizzy drinks, but like, and I know they're bad, and I try not to do it that much but like, I don't really care even though I know they're bad, cause I want it." (FG4, P4)<br><br>"Yeah I definitely have, I know I eat too much sugar, I'm always trying to go on a diet but I usually end up back at square one as I give in too easily." (FG6, P1)
Motivation	The extent of the drive or desire that stimulates behavioural change	"Well for sure the educating the people but sometimes you know no matter how much knowledge you have it's more about changing the behaviour." (I1, P1)<br><br>"It's more about a caring for yourself. Rather than oh I don't want to do that because it's gonna be bad." (I1, P1)

		"Yeah. Um but there is a lot of temptation around us all the time everywhere um and I guess, it's um it's just making small adjustments and, and maybe different choices rather have a piece of fruit, um instead of a biscuit or, something like that." (FG1, P4)<br>"Yeah I agree, there seems to be a sudden health kick in our generation and everyone is trying to be healthier than each other, it's actually quite competitive I guess." (FG6, P2)<br>"I lack the willpower, at the moment to do it. I think this stage of life we're at at the moment, with two young children, where you're constantly tired. And I know, the answer to get natural energy and all the rest of it, is to eat healthily and do exercise. But when you're coming off the back of about two hours sleep, you don't really want those things." (DI1, P2)
Understanding | Acquiring, comprehending and applying insights on sugar, sweeteners and sweet-tasting foods.		
Delivery of Information	How information on sugar, sweeteners or sweet-tasting foods is disseminated and received	"Catchy infographics, I find those really, like impactful advertising, just on visual, you know when they have like heaps of sugar to display to everyone on those stupid diet programs, to be fair that is more impactful than the traffic light system." (FG4, P1)<br>"But, so basically all those packaging, like they mostly use like bright colours like red and stuff. I think that's kind of like sending that sort of, um, yeah. Idea to your brain that that you should be that that is kind of, you know attractive. For the consumer, that's how it's designed." (FG2, P6)<br>"I think schools do quite well, because my kids are a lot more aware of sugar than we ever were when we were kids. They even asked me to put an app on my phone where you can scan food bar codes and it tells you the number of sugar cubes in it." (FG7, P5)<br>"The news and stuff the press has been released about it. But I just know, there is a negative undertone." (DI1, P2)<br>"Where's you said the World Health Organisation uh where, um I mean, you know (pause) presumably the the the broad sheets would have it but where else would you get uh a World Health Organisation message?" (FG3, P1)<br>"I think more of, th-the leaflets visual leaflets like that you know, if if they were put around schools or organisations you know to actually have a visual because, you don't really think about that? When you havin' the drink while you don't realise that necessarily? Um, so it-it makes you realise? It-it's there in your face?" (FG1, P4)<br>"People are heavily influenced by their parents so maybe also (pause) gettin', well yeah. Just you tackle it at every angle I guess. Schools, parents, workplace." (FG1, P1)

		"What was interesting to me was when Jamie Oliver went into schools and he said to the kids choose your lunch. He then said to them before you can eat this you need to burn off the calories around the running track that this food contains. I thought that was brilliant for visualising how much they have got to do to burn off their chosen food." (FG7, P2)<br><br>"The news media pack up on the headline and they don't really fully explain it and everybody just sees the headline and (pause) and and (pause) just changes habits, sometimes unnecessarily." (FG3, P2)<br><br>"How do we get our information and how much time do we spend and where do we get reliable sources about you know just, you have somebody on the internet who's an influencer or whatever and it has in the background a bottle of something uh and you know and there's people looking at that how do we choose our reliable sources to find out about these things so, it takes a lot of time and uh you'll have to look at these things and spend time, really trying to understand." (FG1, P2)
Awareness	A general knowledge of, being conscious about, sensing issues related to sugar, sweeteners or sweet-tasting foods	"When people talk think about sugar, they think about like adding sugar to tea, or you know, coffee and stuff like that maybe, some cookies, cakes, sweet things. And they don't realise how much sugar is in (in overlap) other type of food? So they they, they (pause) you know they- they they eat so much (pause) like, processed food." (FG1, P3)<br><br>"In the sort of the whole process of promoting a balanced diet, then (pause) it's not necessarily, just about (pause) uh cutting down on sugar cane cutting down on coke because it's got too much sugar in it. It's about an awareness sort of. (pause) all the other products that don't look like sugary products." (FG3, P2)<br><br>"Yeah yeah I think people are wisening up because information is out there in a way which is more accessible, not just kinda studies going on behind everyone's back." (FG4, P1)<br><br>"If you had asked me two years ago I would have assumed if it was labelled no added sugar then it would have no sugar in it. It is only more recently that I suspect sweeteners have been added." (FG7, P4)<br><br>"Yeah I have definitely cut down, I put on quite a lot of weight a few years ago and I made a conscious effort to change my diet and I lost quite a lot of weight, it was definitely related to how much sugar I consumed." (FG6, P2)<br><br>"Thing is I feel like I don't really understand it but I always go for low fat or low sugar or no added sugar like I would always go for that if I had a choice." (FG5, P2)<br><br>"But people are so oblivious to it and there's no stuff on the news about it like with kids you need to cut back because parents just don't really realise how bad it really is for children when they just give them sweets and stuff." (FG5, P3)

		"Also I don't think people are aware of the damage that they are doing to their health because there's so much out there, or maybe people just turn a blind eye to it." (FG6, P1)<br><br>"I know that it's generally considered bad for you but if, you wanted me to go into specifics before you answered that truthfully, I couldn't have given you, the specifics other than saying I know it's bad for me on a general level." (DI1, P2)<br><br>"I think instead of preventing it, they should encourage them and then help them see other options other than saying oh this is too expensive you can't buy it now... Cause I've seen it, after Brexit, in some shops (pause) For example there's a certain thing, sausage or whatever I buy, and there's five or ten p difference. People don't realise is just because I always buy that one, I will realise. But I won't know the reason behind it. I will just assume it's Brexit. But it might be, the sugar tax in that sausage. (pause) You don't know." (FG2, P5)
Perception	The way one interprets or regards information related to sugar, sweeteners and sweet-tasting foods.	"But brown rice is better for you so surely brown sugar is." (FG5, P2)<br><br>"It doesn't count! Cause it's a drink!" (FG1, P1)<br><br>"Well the key is, is when they follow it with the word drink… because there's orange juice (pause) drink, then its' like uh oh [it's got something else]." (FG3, P1 & P2)<br><br>"It's a natural fruit. It's a natural sugar! It's natural sugar I think it's different sugar." (FG1, P4)<br><br>"I mean, the general idea of sugar like I know uh glucose, fructose, like um lactose, like all the, um types of… Yeah where you get sugar from. And for example some of them your body doesn't produce and, um so you need to get them for example glucose, and things. But I don't know about the general sweeteners." (FG2, P5)<br><br>"If I have the chance between sugar and honey? Yeah I will choose honey because it's natural." (FG2, P2)<br><br>"Uh, so if you have like (pause) wholemeal food, it contains different types of sugar different types of carbs, (pause) as compared to fruits. Cause th-the fruit, um, they contain a lot of glucose and fructose which are like, simple sugars?... i-in fruit you-you've got th-th-the type of sugar that you actually use quite quickly?... So uh (pause) as compared to, let's say, whole-grain, um (pause) pasta or whatever, which also contains a lot of (pause) carb, that's that is a different type… from what I know, it's for your health, it's better to eat vegetables, rather than fruits? Or grains? If you if you need carbs. If you need this type of uh (pause) thing. It's better to eat grains or y'know cereals, without, added sugar. Uh, rather than fruit?" (FG1, P3)<br><br>"Oh yeah white is so bad for you, they have to bleach it and put sugar in it." (FG5, P2)

Proficiency	The deeper knowledge and expertise in matters related to sugar, sweeteners or sweet-tasting foods	"Thing is they're always like no added sugar but I didn't realise as soon as you blend fruit that your body treats it as white sugar, out the packet, and also like if you made a smoothie with like two bananas, one apple, that would only count as one of your five a day, as soon as you blend it the sugar becomes free radicals or something and your body just treats it as sugar." (FG5, P2)<br><br>"It's a lot of lack of education in, like, knowing that actually if you, cook a tomato sauce or something like the sugars within the tomato, or you know there's a, the fruit and food and vegetables have, naturally occurring stuff in them. So you don't actually need to add, anything to it. So you will still get a feeling of like oh like in a cake you could add some banana or something, and you get some sweetness instead of (laughs) instead of adding sugar you know there's lots of things like that." (DI1, P1)<br><br>"The addition of sugar on proteins (inaudible). You know, um help cancer cells to, (sigh) um metabolise certain uh things such as collagen for example you know when if the cancer cells can degrade collagen better. They um, they can go from one tissue to another so that's uh, that's for example. An example of study." (I1, P1)<br><br>"People just don't realise it's not only about like, sugar cubes or something like that." (FG1, P3)<br><br>"I feel like coke is the drink where people are very educated on, like everyone knows it's bad." (FG5, P3)<br><br>"I think a lot has been done to educate people on sugar, but there seems to be no education on sweeteners and what they are." (FG7, P4)
Reasoning	To apply logic while processing information, in order to form inferences about sugar, sweeteners or sweet-tasting foods	"There's a lot of messages around and I think now, there's more of an emphasis on (pause) people (pause) lookin' at it thinking right what do I do? But th-then some people think that'll never happen to me. Whoosh! Shall eat what I want." (FG3, P1)<br><br>"Yeah like I don't know what to believe any more because there are so many of this fad diets and all that I like don't know who to trust." (FG5, P4)<br><br>"I think you might be replacing one bad thing with another bad thing!" (FG7, P1)<br><br>"I think my concern would be if people, mis-interpreted the message that said sweeteners are okay, and sugars are less okay. People might think, well I won't bother exercising now and they think then if if I just turn to sweeteners." (FG3, P1)<br><br>"I would buy, the normal one? But just eat less? Probably? So if if I if I (pause) did buy, a lite version of something I would probably (pause) be thinking that okay I can eat more because it's lite?" (FG1, P1)

		"How do we get our information and how much time do we spend and where do we get reliable sources about you know just, you have somebody on the internet who's an influencer or whatever and it has in the background a bottle of something uh and you know and there's people looking at that how do we choose our reliable sources to find out about these things so, it takes a lot of time and uh you'll have to look at these things and spend time, really trying to understand." (FG1, P2)<br>"I-I think it's something complex debate about, just because something technically could be called natural whether or not that it is it's actually is more beneficial (pause) than something that was (pause) i-in effect (pause) cooked up in a lab, that doesn't necessary follow that that's better than that, simply. Bu-but it's quite a complex matter." (FG1, P2)<br>"I think maybe if you suffer from diabetes or something like that there are possibly some sweeteners that are better for blood sugar regulation, I don't really know. Yeah, I think that would maybe be an advantage, but from my point of view, someone that doesn't have a condition, I don't see any advantage." (FG7, P3)<br>"But is this being considered? Because what now what I'm thinking is, um obviously uh there're other stuff that are quite (pause) you know, not so beneficial even harmful, uh in the shops. So it's not just bout sugar, but if we actually, go for this, these regulations in terms of what every single thing we eat (pause) then it would be the end of an era. Of marketing and brands and everything." (FG2, P6)
It's Not Up to Me | One takes a passive approach towards sugar, sweeteners and sweet-tasting foods, because intake is subject to other factors.		
Beyond Individual Control	Factors beyond the control of an individual, including external influences	"They probably just have one, so they make that choice for you really, in terms of sweeteners." (FG1, P4)<br>"Fair enough if you're big and you're top of reasonable BMI and that's the way, like I know someone who's got a thyroid problem and it doesn't matter how clean she eats she will put on weight and she'll go running every week but she's still a big woman and she can't help it, probably really depressing and crappy on their self-esteem." (FG4, P1)<br>"But also, it depends on your, previous experiences. For example. Up your upbringings, what they drink what they eat. And then you see what they eat and you do the same so." (FG2, P5)<br>"Yeah as a kid I had way more sugar than my children have now. Every time I was picked up from school or play group my mum would give me a bag of sweets. Golden syrup on my porridge." (FG7, P3)<br>"I think we're lucky in this country as well cause I've travelled a lot and m- orange juice for example over here (pause) if it says natural, pure orange juice it is a hundred percent pure orange juice in America for example, you

		try finding orange juice, That doesn't have added sugar (pause) it used to be very hard they are getting better but they used to be yeah. Not available!" (FG3, P3)<br>"Yeah but at the same time you might want baked beans but you're not asking for all the extra added sugar which has gotten has increased drastically over the years like it didn't used to have that much sugar in it, and you can't really get ones without the enormous amount of sugar in it and that's not necessarily their fault, baked beans are supposed to be healthy for you, it's the manufacturers' fault for putting that much in there in the first place." (FG5, P1)<br>"I think the nation is starting to become a lot healthier in general, like I'll admit I used to be someone who would always buy a fizzy drink, thinking about it now I don't really know why but I always did, now I rarely even look at them and I usually buy water." (FG6, P1)<br>"I think people need professional help! You know for sugar? Cause of the fact that I've I- I- y-yeah. I think she's right. It is a drug (pause) and when I when I need, I need it." (FG2, P6)<br>"I think the trouble is, I only noticed it when I went on a sugar-free diet, is that you don't realise how accustomed your palate is to sweetness. So with ketchup and other things like that you wouldn't imagine are loaded with sugar they are. You don't realise how your palate has become accustomed to such a sweet taste in everything and then when you remove the sugar completely everything becomes so bland." (FG7, P2)
Strategies and Regulations	Legislations; official large-scale measures put in place	"I think some places they (pause) cause it's less (pause) you sort of automatically get th-the diet? Because it's cheaper? As opposed to getting I think if you asked for, a coke they almost I-I don't know how true this is." (FG1, P1)<br>"You know, if it's really a health issue problem, it has to be you know, peep-people can't take responsibility most of the time, so you know that's, that's a lot to do with that! You know, teaching people how to take responsibilities… But, at the end of the day, you know, if that doesn't work, is like people are children you know, you have to tell them off and the only way is punishment! Isn't it?" (I1, P1)<br>"It's a government's responsibility to make sure companies are in like, and if there's a UK health crisis aa best method of treating best method of disease or not is prevention, so you need to, you need to set the standard, you can't just rely on people to know what's best for them." (FG4, P1)<br>"IF there were more taxes which manufacturers had to fork out for then maybe they'd actually stop filling their products with so much sugar, that's the message which is trying to be achieved at the end of the day." (FG6, P2)

Deception	The idea of traps, tricks or temptations by manufacturers; distrust of food industry	"I think people don't really think about what they're putting in their shopping baskets, there's so many brands of the same stuff and so many adverts, it's easy to fall into the trap of buying things rather than sticking to a plan/shopping list." (FG6, P2)<br>"I think it's sneaky how much they put in stuff, it can be hard to stick to your plan or keep things in moderation when companies load things with sugar and fat." (FG6, P3)<br>"Some big companies are being um getting paid by some of the big companies to the doctors and they are giving kind of (pause) prejudice or kind of biased advice? So you shouldn't always trust the professionals as well. I just like, do some research, ask people, what are their opinion, and stuff. So don't just go to professionals." (FG2, P5)<br>"Well I'm a cynic with this kinda stuff and I work in marketing. So (pause) y'know, I know first-hand, that, it is spin city you know? Everything is being spun. So it's just that oh no sugar, uhhuh? What else is in it then? Because I'm tasting sweetness somewhere, and unless you're some kind of magician, you've put something in it to make it sweet, so you saying no sugar it's just like okay, well how're you harming me (pause) somewhere else?" (DI1, P2)<br>"Cause obviously they're not allowed to lie on the tables but I feel like they deliberately make it confusing cause obviously it's in their interest that you don't understand." (FG5, P2)<br>"I feel tricked, how much was there already if there's no added sugar." (FG5, P4)

Table S2. Moderator Guide.

Questions will focus on participants' beliefs about sugar and sweeteners, which would you choose to consume and why, your considerations towards natural versus artificial versus synthetic and non-caloric versus low-calorie sweeteners, your thoughts on reducing sugar intake versus sweetness intake, as well as your opinions on current and potential strategies to reduce free sugar intake.

Opening
- Everyone to introduce yourselves: name and favourite dessert
- Tell me the most recent time you consumed a memorable dessert
- What came to your mind immediately when I asked about 'sweet foods' or 'desserts'?

Introduction
- Which type of drink is your favourite and why?
  - Regular soft drink, diet soft drink, juice, etc.
- When you consume foods and drinks, do you usually consume with sugar or with sweeteners? Why?

Sugar
- Pertaining to sugar, can you share with me what factor(s) will drive you to choose to consume sugar over sweeteners?
- What are the concerns, you have about consuming foods or drinks with sugar?
  - Do you think there are any advantages or disadvantages to consuming foods or drinks with sugar?
- Have you ever thought about cutting down on sugar? Why?
- Are you aware of the health reasons for cutting down on sugar?
  - Obesity?
  - Diabetes?
  - Tooth decay and loss?
  - Will you cut down on sugar consumption for the above reasons?
- If you had to provide a rough estimate, what do you think is the Public Health England recommended daily sugar allowance?
- 30 grams is the correct answer, which is roughly 7 sugar cubes. A single can of Coca-Cola contains 35 grams/ a 360ml bottle of Innocent 'Spark' smoothie contains 43.2grams/ a Starbucks signature hot chocolate in venti size contains 60grams *(refer to print-out).* How does that make you feel knowing it is about twice over the recommended allowance?
  - Does this new knowledge make you want to drink less of these sugar-sweetened drinks?
- A recent report stated that 2017 was the first time that bottled water sales have been bigger than Coca Cola sales. Why do you think people are switching to a water option?
  - Will you switch to a water option? Are there other options you will switch to?

(Time check: we have completed the section on sugar, we have three more sections to go ☺)

Sweetener

- Would you go for a bottle of water or diet soft drink? Why?
- Are you familiar with the variety of sweeteners currently available? *(refer to print-out)*
    - Natural versus artificial versus synthetic sweeteners, do you know what they are? What they are made from?
    - Do you know that there are non-caloric and also low-calorie sweeteners? Do you know which ones are which?
- Can you share with me what factor(s) will drive you to choose to consume sweeteners over sugar?
- What are the concerns you have, about consuming foods or drinks with sweeteners?
    - Do you think there are any advantages or disadvantages to consuming foods or drinks with sweeteners?
    - Do you ever consider potential health issues which could arise from long-term consumption of sweeteners? (Artificial or synthetic sweeteners?) If so, what?
- Do you consider artificial versus natural versus synthetic, non-caloric versus low-calorie sweeteners differently? How so?
    - Are you more concerned with one type over another?
    - Are you more motivated to consume one type over another?

Sweetness

- Given that both sugar and sweeteners seem to have their own sets of issues, do you think we should reduce the amount of sugar in our diet, or reduce the amount of sweet foods and drinks (regardless of source of sweetness: sugar or sweetener) in our diet?
- Do you think you will face difficulty in reducing sweetness in your diet?

(Time check: we are now down to the final section on strategies ☺)

Strategies

- Going into reducing the amount of sugar in our diet, what do you think would be the best method for trying to reduce sugar consumption?
    - Product labelling like traffic light system or "low sugar"? Do you trust labels?
    - Scare tactics? *(refer to print-out)*
    - Regulations similar to those placed on cigarettes and alcohol?
    - Education? Particularly in schools and/or workplace?
- The sugar tax is one method. "A 20% tax on sugary drinks would prevent 3.7 million people becoming obese in the UK over the next decade." *(refer to print-out)* What are your initial thoughts when hearing this quote?
    - What effect do you think this will have on the consumption of sugary and sweetener products?
    - Do you think it should be manufacturers or consumers that should pay the soft drinks tax? Why?
- Chocolate bars are shrinking in size and drink manufacturers are reducing their sugar amount due to the taxes, have you personally noticed these changes when consuming these products and what effect has it had on your shopping behaviour?
- How do the words 'diet' or 'zero calories' impact your choice of beverage?

- Do you think strategies/policies/recommendations should be about replacing sugar with sweeteners?
    - Earlier on, some of you think that we should only reduce sugar intake, while some of you think that we should reduce sweetness in the diet, regardless of source of sweetness. What societal-changes or methods do you think will be beneficial?

Conclusion

- Of all the things we discussed, what to you is the most important factor in deciding to consume sugar or sweetener?
    - If health, which aspect of health (illness like diabetes, or weight management, or dental health etc.)?
- With all things considered (like taste, health, accessibility etc.), which would you prefer to consume the most and least?
    - if sweetener – specify natural/artificial/synthetic/non-caloric/low-calorie
- *(Give a brief oral summary)* Is this an adequate summary?
- Is there anything which has not been discussed that you feel strongly about and would like to bring up now?
- Can you share with me why you decided to participate in this focus group?

Thank you very much for all your input.
